# Computational Modeling of Hepatitis C Virus Envelope Glycoprotein Structure and Recognition

**DOI:** 10.3389/fimmu.2018.01117

**Published:** 2018-05-28

**Authors:** Johnathan D. Guest, Brian G. Pierce

**Affiliations:** ^1^Institute for Bioscience and Biotechnology Research, University of Maryland, Rockville, MD, United States; ^2^Department of Cell Biology and Molecular Genetics, University of Maryland, College Park, MD, United States

**Keywords:** hepatitis C virus, vaccines, modeling, design, E1E2, glycoproteins, antibodies

## Abstract

Hepatitis C virus (HCV) is a major global health concern, and though therapeutic options have improved, no vaccine is available despite decades of research. As HCV can rapidly mutate to evade the immune response, an effective HCV vaccine must rely on identification and characterization of sites critical for broad immune protection and viral neutralization. This knowledge depends on structural and mechanistic insights of the E1 and E2 envelope glycoproteins, which assemble as a heterodimer on the surface of the virion, engage coreceptors during host cell entry, and are the primary targets of antibodies. Due to the challenges in determining experimental structures, structural information on E1 and E2 and their interaction is relatively limited, providing opportunities to model the structures, interactions, and dynamics of these proteins. This review highlights efforts to model the E2 glycoprotein structure, the assembly of the functional E1E2 heterodimer, the structure and binding of human coreceptors, and recognition by key neutralizing antibodies. We also discuss a comparison of recently described models of full E1E2 heterodimer structures, a simulation of the dynamics of key epitope sites, and modeling glycosylation. These modeling efforts provide useful mechanistic hypotheses for further experimental studies of HCV envelope assembly, recognition, and viral fitness, and underscore the benefit of combining experimental and computational modeling approaches to reveal new insights. Additionally, computational design approaches have produced promising candidates for epitope-based vaccine immunogens that specifically target key epitopes, providing a possible avenue to optimize HCV vaccines versus using native glycoproteins. Advancing knowledge of HCV envelope structure and immune recognition is highly applicable toward the development of an effective vaccine for HCV and can provide lessons and insights relevant to modeling and characterizing other viruses.

## Introduction

Hepatitis C virus (HCV) is estimated to have infected over 70 million globally, with millions of new cases every year (1). Chronic HCV infection can lead to cirrhosis and hepatocellular carcinoma (HCC) and deaths due to HCV are rising worldwide (1). In the United States, the yearly rate of deaths resulting from HCV infection has surpassed that of human immunodeficiency virus (HIV) and other infectious diseases (2). Direct-acting antivirals (DAA) for treatment of HCV infection have high cure rates, but face major issues: limited patient accessibility due to high costs of treatment (3), little to no awareness of infection in most HCV-positive individuals (4), and neither prevention of reinfection (5) nor elimination of HCC risk (6) in cleared HCV patients following DAA treatments. Thus, there is an ongoing major need for an effective prophylactic vaccine for HCV in order to greatly reduce global disease burden (4, 7).

A major barrier to vaccine and targeted therapeutic efforts is the high sequence variability of HCV, as exemplified by its seven confirmed genotypes, which are subdivided into 86 confirmed subtypes as of June 2017 (8) that can differ by greater than 15% in sequence (9). Furthermore, HCV rapidly mutates to form quasispecies within infected individuals, permitting active escape from neutralizing antibodies; this mechanism was clearly demonstrated in a clinical trial of monoclonal antibody HCV therapy followed by deep sequencing of HCV in patients (10, 11). Effective targeting of this diverse virus would be greatly facilitated by a detailed understanding of the molecular determinants of viral fitness, assembly, and function (12).

**Figure 1:**
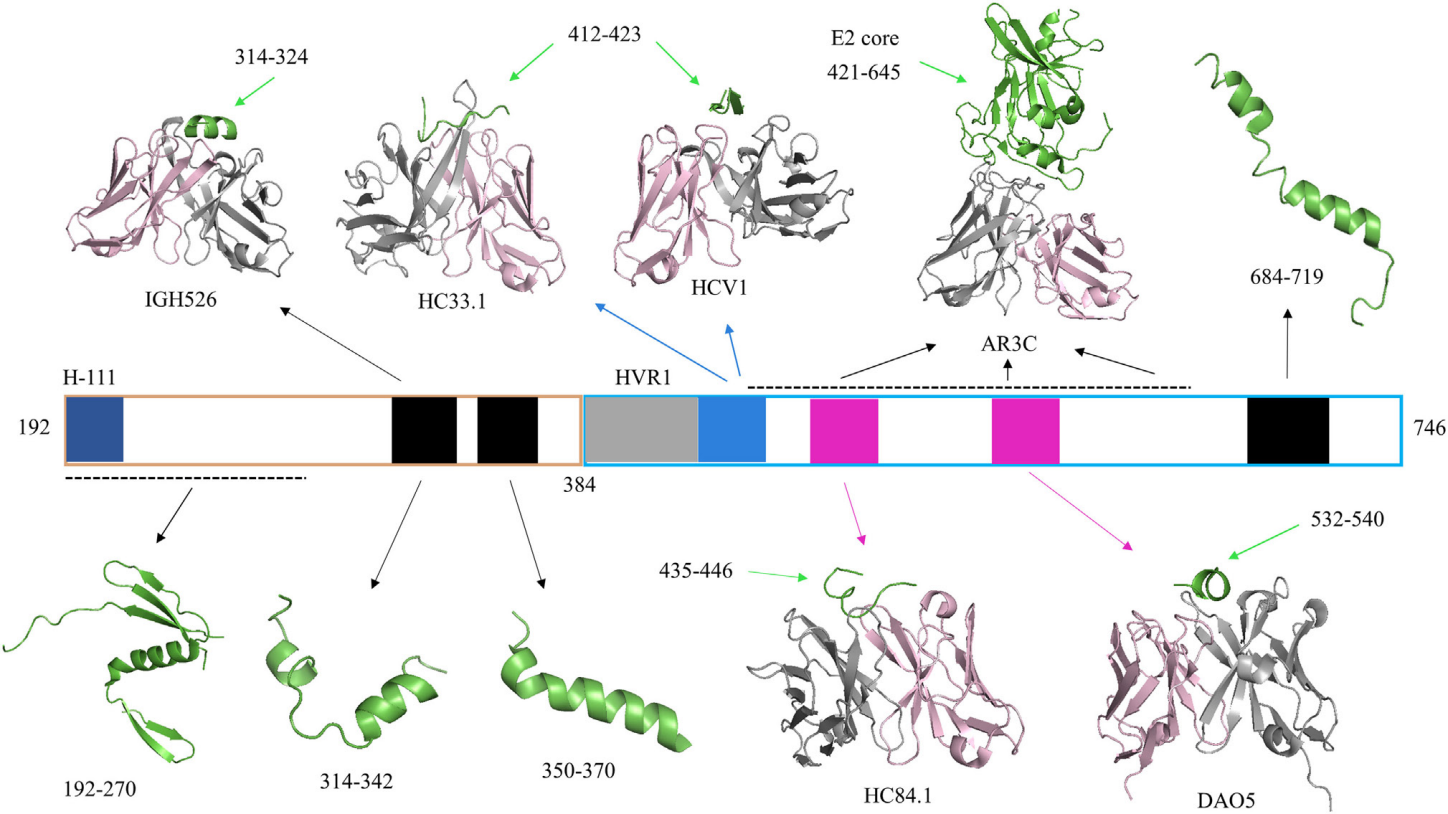
Representative crystallographic and NMR structures of E1E2 regions. Hepatitis C virus (HCV) peptides and proteins in all structures are colored green. Antibody heavy chains are colored gray and light chains are colored light pink. PDB codes of representative structures are 4UOI (192–270), 4N0Y (314–324/IGH526), 2KNU (314–342), 1EMZ (350–370), 4XVJ (412–423/HC33.1), 4DGY (412–423/HCV1), 4MWF (421–645/AR3C), 4JZN (435–446/HC84.1), 5NPJ (532–540/DAO5), and 2KZQ (684–719). Residue ranges of the E1E2 sequence corresponding to specific sites are highlighted by colored bars for reference: H-111 epitope at N-terminus of E1 (aa 192–202, dark blue), E2 hypervariable region 1 (aa 384–410, gray), Domain E (aa 412–423, blue), Domain D/AR3 (aa 434–446, magenta), and Domain B/AR3 (aa 529–535, magenta). Other regions of E1 and E2 with corresponding structures are shown in black bars, and black dashed lines represent gaps in a crystal structure.

The envelope glycoproteins E1 and E2 are targets of anti-HCV antibodies (13), and have been used in numerous B cell vaccine development efforts (14–18) and several clinical trials (19, 20) [reviewed by Fauvelle et al. (21)]. Epitope mapping and other characterization efforts have classified E2 antibody epitopes into five antigenic domains (A–E) (22), a nomenclature that will be used in this review. Alternative definitions such as antigenic regions (antigenic regions 1–3) (23) and epitopes I–III (24) have been used to identify these regions on the E2 surface, in addition to epitopes on E1E2 (antigenic regions 4–5) (25). Despite advances from numerous epitope mapping studies, the overall structure of these glycoproteins and the structural basis of neutralizing antibody engagement of many key epitopes have yet to be determined experimentally. Some structures representing portions of these proteins have been determined to date, spanning a conserved “core” region of E2, portions of E1, and multiple mAb-bound E1 and E2 peptides (Figure 1; Table 1). In contrast, other highly variable viruses, such as HIV and influenza, have likewise been longstanding targets of vaccine design efforts, and the assembly of their envelope glycoproteins, hemagglutinin (HA), and Env have been determined at high resolution (26, 27). Additionally, there are many HA and Env neutralizing antibodies structurally characterized in complex with their epitopes (28–30), providing insights that enabled a number of successful structure-based vaccine design efforts (31–34). Given the relatively limited availability of HCV structural data, as well as the challenges for experimental structure determination presented by innate flexibility (22, 35, 36) and high glycosylation (37) of HCV glycoproteins, there is a major opportunity to bridge gaps in knowledge of current structural and mapping data through computational structural modeling, enabling a comprehensive view of glycoprotein structure, recognition, and dynamics.

**Table 1:** Experimentally determined structures of E1, E2, and monoclonal antibodies.

Structure code^a^	Hepatitis C virus (HCV) glycoprotein^b^	Residue range^c^	Antibody	Reference
**X-ray crystallography**				
4UOI	E1	192–270	–	(38)
4N0Y	E1	314–324	IGH526	(39)
4GAG	E2	411–424	AP33	(40)
4GAJ	E2	412–423	AP33	(40)
4GAY	E2	Unbound mAb	AP33	(40)
4DGY, 4DGV	E2	412–423	HCV1	(41)
4G6A	E2	412–423	AP33	(42)
4HS6	E2	412–423	MRCT10.362	(43)
4HS8	E2	412–423	hu5B3.v3	(43)
4WHT, 4WHY	E2	412–423	3/11	(35)
4XVJ	E2	412–423	HC33.1	(44)
5FGB	E2	417–421	HC33.4	(45)
5FGC	E2	415–423	HC33.8	(45)
5EOC	E2	412–422^d^	C2	(16)
5KZP	E2	412–423^d^	HCV1	(17)
5VXR	E2	412–423	MAb24	(46)
4MWF	E2	421–645^e^	AR3C	(47)
4WEB	E2	486–645	2A12	(48)
4Q0X	E2	434–442	mAb#12	(49)
4HZL	E2	430–442	mAb#8	(50)
4JZN	E2	435–446	HC84.1	(51)
4JZO	E2	436–446	HC84.27	(51)
5ERW	E2	438–446	HC84.26	–^f^
5ESA	E2	Unbound mAb	HC84.26	–^f^
4Z0X	E2	435–446	HC84.26.5D	(52)
5NPH, 5NPI, 5NPJ	E2	532–540	DAO5	(53)
3U6R	E2	Unbound mAb	1:7	(54)
4JVP	E2	Unbound nanobody	D03	(55)
**Nuclear magnetic resonance**				
1EMZ	E1	350–370	–	(56)
2KNU	E1	314–342	–	(57)
2KZQ	E2	684–719	–	(58)
**Electron microscopy^g^**				
5759	E2	384–717	AR3A	(47)
5760	E2	384–717	AR3A, AR2A	(47)
5761	E2	384–717	AR2A, CD81	(47)
8338, 8339, 8340	E2	412–645	AR1B, AR2A, HCV1	(36)

*^a^Protein Data Bank (59) or EMDataBank (60) codes shown. Multiple codes are shown in cases with multiple entries reported from same study containing the same residue range and binding partner(s), corresponding to different crystallographic symmetry forms, electron microscopy reconstructions, or HCV isolate sequences*.

*^b^In the case of unbound antibody, glycoprotein target of antibody is given for reference*.

*^c^Residue numbering based on H77 isolate. For crystallographic structures, range reflects resolved residues present in coordinates*.

*^d^Cyclic epitope-based designs are present in these structures*.

*^e^This E2 core construct included engineered deletions of residues*.

*^f^The coordinates for these X-ray structures have been released in the PDB (59) but have no publications associated with them*.

*^g^These negative stain electron microscopy structures have resolutions of 16–30 Å, thus provide approximate envelopes for fitting high-resolution crystallographic or modeled structures*.

This review provides an overview of efforts to model HCV envelope structure and recognition, which have collectively yielded many valuable insights into this virus. These efforts include initial work to model the E2 structure, recent modeling of the full-length E1E2 heterodimer, and modeling focused on other aspects of HCV, such as the dynamics of epitopes and recognition of antibodies or coreceptors; a subset of these studies is summarized in Table 2. Models and hypotheses from these studies can be used to inform future experimental and computational modeling efforts, as well as structure-based design of effective vaccines.

**Table 2:** Representative modeling studies of hepatitis C virus envelope glycoproteins and receptors.

Target	Model	Methods^a^	Year	Reference
E2	Structure	Homology-based modeling	2000	(61)
E2	Structure	Homology-based modeling, disulfide mapping	2010	(62)
E2-CD81	Complex structure	Restraints-guided docking	2013	(47)
E2	Front layer dynamics	Molecular dynamics simulation	2016	(36)
E1E2 transmembrane	E1 trimerization, E1E2 heterohexamer	Docking with restraints	2015	(63)
E1E2	Structure	Evolutionary constraints-based structure prediction, homology-based modeling, experimental mapping residue constraints	2017	(64)
E1E2	Structure, high order assembly	Homology-based modeling, *ab initio* structure prediction, experimental mapping residue constraints, docking	2017	(65)
SR-BI	Structure	Homology-based modeling	2013	(66)
CD81-Claudin	Structure	Homology-based modeling, docking	2012	(67)

*^a^Summary of modeling methods used*.

## Models of the E2 Structure

Prior to experimentally determined structures of the E2 glycoprotein, computational models were developed to predict its tertiary and quaternary assembly. These efforts used structures of flavivirus and alphavirus class II fusion proteins as modeling templates (61, 62). A crystal structure of the E2 glycoprotein of tick-borne encephalitis virus (PDB code 1SVB) (68) served as the main template for the first of these modeling studies, which was reported over 15 years ago (61). The authors predicted that E2 assembles into an elongated monomer and also described putative E2 homodimerization and a possible site of interaction with E1. Further analysis of this model found that the binding regions predicted for CD81 and multiple E2 mAbs were exposed epitopes on the modeled E2 surface. A more recent E2 modeling study was largely based on the structure of the Semliki Forest virus E1 glycoprotein (PDB code 2ALA) (69), with particular emphasis on shared secondary structure elements, and incorporated nine experimentally determined E2 disulfide bonds as modeling constraints (62). The resulting model included three predicted domains for E2, with domain I (the first in order of amino acid sequence) corresponding to a β-sandwich positioned between the other two domains and forming a tightly packed CD81-binding site that roughly corresponds to antigenic domains B, D, and E. As noted by the authors of the latter modeling study (62), these two E2 models are divergent in several regards, including their predicted disulfide bonds, predicted E2 oligomerization and degree of coverage of the E2 glycoprotein. Subsequent X-ray crystallographic determination of two E2 core crystal structures revealed features distinct from structurally characterized class II fusion proteins (70, 71), including more compactness than the classical three domain organization of class II fusion proteins, despite retaining its immunoglobulin β-sandwich domain (47). Overall differences in architecture presented a likely impediment to template-based modeling, notwithstanding potentially accurate prediction of certain features and secondary structure elements. Regardless, these E2 modeling studies were important first steps in characterizing HCV glycoproteins, providing useful testable hypotheses in the absence of an experimentally determined E2 structure.

## Models of E1E2 Assembly

Currently, no experimentally determined structure is available for the E1E2 complex, which has led to two recent studies that have presented structural models of this assembly (64, 65). For clarity, they will be referred to as E1E2-C and E1E2-F, after their respective first authors. A third E1E2 model has been proposed, but does not contain a complete heterodimer and, therefore, will not be discussed in detail (72). The E1E2-F and E1E2-C models were generated using distinct methodologies. The E1E2-C model was generated through mapping antibody epitopes with shotgun mutagenesis (73), residue contact prediction with evolutionary coupling analysis (74) supplemented by known contacts of the E2 core crystal structure (47), as well as β-sheet pairing predictions using the bbcontacts algorithm (75). The final E1E2-C model of the heterodimer was generated using the CNS suite (76) and a distance geometry simulated annealing protocol. The E1E2-F model was likewise generated using a detailed computational pipeline, while also ensuring that the model corroborated previous experimental findings. Prediction of the E1 structure combined a partial crystal structure of E1 (38) with structural homolog phosphatidylcholine transfer protein (PDB code: 1LN2) (77) in the Molecular Operating Environment program (78). E2 was modeled in the Robetta server (http://robetta.bakerlab.org/), which added missing loops and termini to the E2 core crystal structure. Following *ab initio* prediction and molecular dynamics (MD) simulations of E1 and E2 transmembrane regions (TMs), RosettaDock (79) was used to dock the E1 and E2 models to predict their heterodimeric assembly, followed by symmetric docking of the E1E2 model to form heterohexameric E1E2 models (trimers of E1E2).

**Figure 2:**
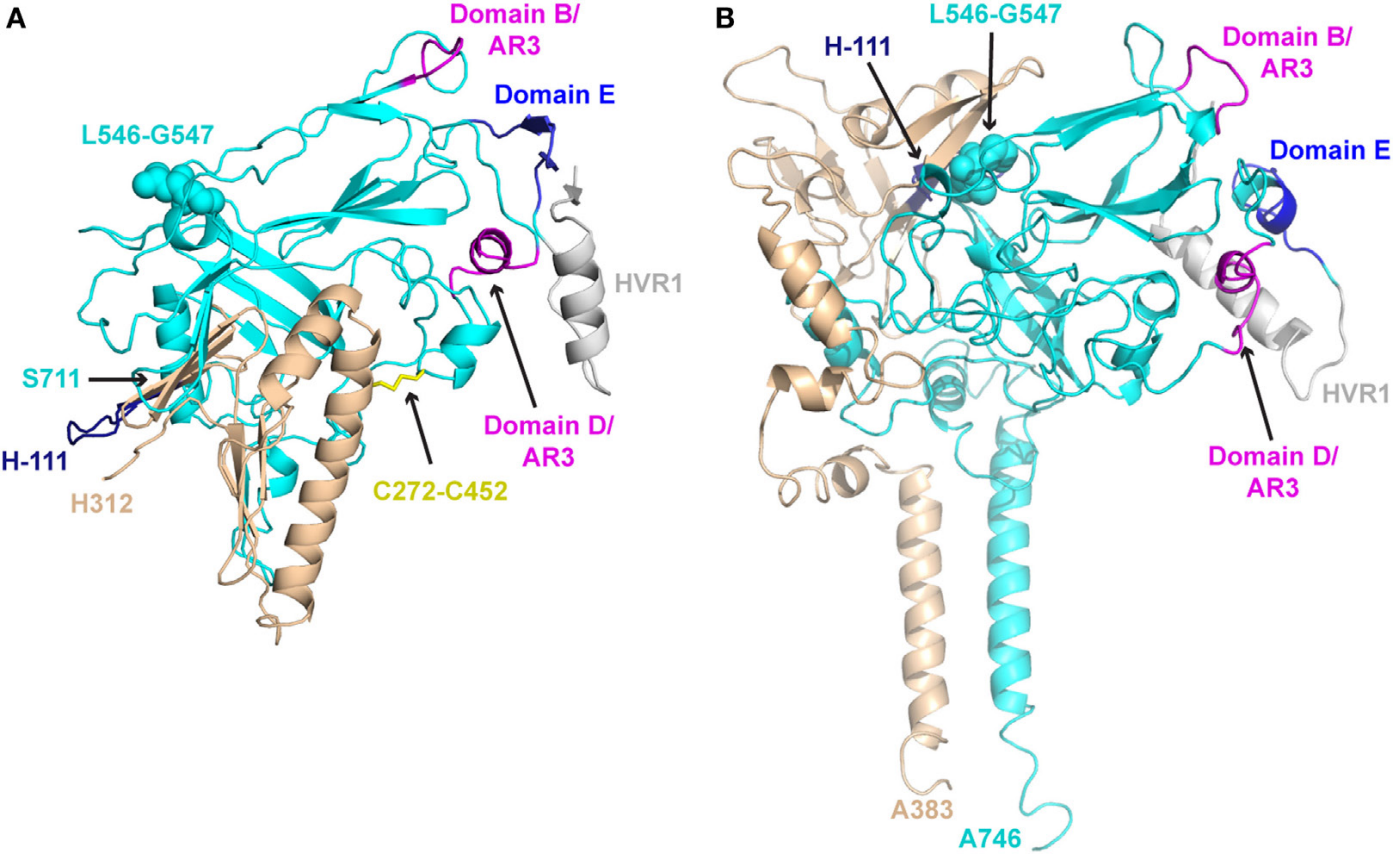
Structural models of E1E2 heterodimeric assembly. **(A)** E1E2 model from Castelli et al. (E1E2-C) (64) in comparison with **(B)** E1E2 model from Freedman et al. (E1E2-F) (65), oriented in the same frame of reference based on E2 core regions. E1 and E2 glycoproteins are shown as tan and cyan cartoons, respectively, while key epitopes are colored and labeled, as in Figure 1: H-111 epitope at N-terminus of E1 (“H-111,” aa 192–202, dark blue), E2 hypervariable region 1 (HVR1, aa 384–410, gray), Domain E (aa 412–423, blue), Domain D/AR3 (aa 434–446, magenta), Domain B/AR3 (aa 529–535, magenta). Additionally, selected features of modeled E1E2 are highlighted: the predicted E1–E2 disulfide bond of E1E2-C (C272–C452), shown as yellow sticks, and E2 residues L546–G547, predicted to interact with E1 in E1E2-F model, are shown in spacefill on both models. C-terminal residues of E1 and E2 are also labeled for both models (H312, S711 for E1E2-C, A383, A746 for E1E2-F).

Comparison of the E1E2-C and E1E2-F models reveals some similarities, but also major distinctions between them (Figure 2). Unsurprisingly, the E2 core region is mostly conserved between the two models, as both E1E2-C and E1E2-F incorporated residue contacts from existing E2 core structures. This conservation includes the overall arrangement of antigenic domains B, D, and E. However, the quaternary structure of the two models display striking differences, with a dramatic change of E1 orientation relative to E2. One notable difference is an inter-chain disulfide bond at C272–C452, which is proposed by E1E2-C on the basis of their antibody epitope mapping data, but is not present in E1E2-F. Additionally, E2 residues 546–547, which are associated with antigenic domain C as well as E1E2 mAb binding based on global epitope mapping studies (80, 81), are located at the predicted interface with E1 in E1E2-F but not E1E2-C. This site has been associated with E1E2 assembly in a recent screening effort, which found that a peptide from JFH-1 (aa 546–560 based on H77 numbering) inhibited HCV entry and bound E1E2 (82). Finally, there are differences in model coverage of E1 and E2 (E1E2-F represents the full glycoprotein sequences), as well as the conformations and orientations of the flexible region at the N-terminus of E2 (HVR1 and antigenic domain E). These models offer intriguing possible modes of E1E2 heterodimerization, providing an avenue to potentially design stabilized vaccines in the absence of an experimentally determined structure, and future studies can confirm (e.g., through structure-guided mutagenesis of predicted interface residues) or refine these models.

## Recent Modeling Studies of E1 and E2

Other studies have used existing crystal structures to explore conformational flexibility and assembly, capturing the dynamic properties of E2. Flexibility of the CD81-binding site (CD81bs) has been examined in a recent study using MD simulations, hydrogen–deuterium exchange (HDX), and calorimetry (36). The MD simulations suggested that the helical region near residue 434 displays a pronounced tendency to “drift” away from the E2 core, which is supported by crystallographic studies of multiple antibodies bound to the corresponding epitope of the peptide (49). Mobility of these regions has also been examined using an E2 core crystal structure plus modeled domain E, finding a broad range of conformations that occasionally resembled those observed in X-ray structures of the antibody-domain E complex (83).

Studies focused on modeling E1E2 TM domains have provided insights into determinants of E1E2 heterodimerization and assembly. Following descriptions of SDS-resistant E1E2 TM heterodimers and E1 trimers, a trimeric model of E1 TM domains was generated (63). This model was partially based on an experimentally determined structure of the monomeric E1 TM (PDB code: 1EMZ) (56) and also included constraints to enforce putative inter-helical interactions between G_354_xxxG_358_ residues, a motif essential for E1 TM assembly and conserved in other helix–helix interactions (84). Critical charged and polar residues were exposed in the trimeric model, allowing E1 trimers to form key interactions with E2 such as the putative K370–D728 salt bridge, which was also observed in a separate study that performed MD simulations of the E1E2 TM heterodimer (85, 86). These studies and others (87, 88) have used modeling on this small yet critical region to gain a clearer picture of E1E2 association.

In combination with experimental mutagenesis data, modeling has been used to explore how residue substitutions affect glycoprotein stability and structural integrity. Using the program Rosetta, *in silico* alanine mutagenesis of all E2 residues available in one of the E2 core crystal structures predicted changes in protein stability for each mutant (80). Alanine mutants with greatest predicted destabilizing effects on E2 corresponded to those with experimentally measured loss of binding for 14 conformationally sensitive HCV mAbs during global alanine scanning mutagenesis of E2. In the same study, alanine scanning data from each mAb was analyzed by hierarchical clustering to form groups of residues that delineated energetically linked regions on the E2 surface and core. These studies highlight how the incorporation of experimental mutagenesis data and other techniques (e.g., HDX) with modeling methods can reveal key aspects of glycoprotein flexibility and structural determinants.

## Modeling Antibody Recognition

Modeling conserved epitopes of HCV glycoproteins has been valuable for elucidating the structural basis of broadly neutralizing antibody (bnAb) recognition. Crystal structures for the domain E peptide (E2 residues 412–423) bound to HCV1 (41), HC33.1 (44), 3/11 (35), and AP33 (40, 42) established different conformations of the same conserved epitope. Understanding the structural basis of these variable conformations was critical for determining why rare domain E mutations evaded neutralization by some of these antibodies, but not all (43, 89). Computational alanine scanning of antigenic domain E bound to HC33.1 predicted a decrease in antibody affinity when key binding residues were mutated, but no change in affinity when a “glycan shift” viral escape mutation was modeled (44). The program GlyProt (90) was used to model E2 glycosylation in the HCV1 and HC33.1 complexes, showing that glycosylated N415 in domain E would be sterically unfavorable for binding by HCV1, which like AP33 engages the β hairpin form of the epitope, but it would be permitted at the exposed N415 residue in the extended conformation bound by HC33.1 (44). Additional modeling of domain E structures in the same study used the PEP-FOLD server (91) to generate *ab initio* peptide models that largely matched a β-hairpin conformation, suggesting that this folding pattern is preferred for domain E in the absence of antibody engagement and that this conformation can be disrupted by several domain E mAbs (35, 44). Computational mutagenesis and modeling not only helped to delineate domain E antibody recognition, but also domain D recognition by an affinity-matured antibody (52). These techniques can be used to build on structural knowledge of other antibody epitopes to E1, E2, or the E1E2 heterodimer, especially if similar crystal structures of antibody–antigen complexes provide informative comparisons.

## Modeling Receptor Structure and Recognition

**Figure 3:**
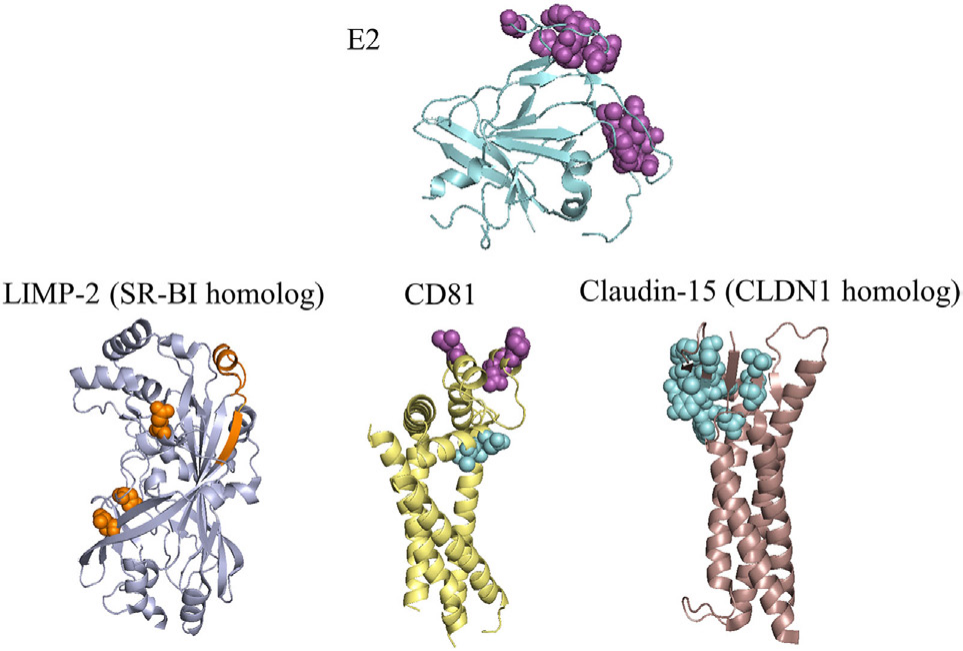
Residues of E2 and coreceptors that influence hepatitis C virus (HCV) entry and infection. E2 and three receptors are depicted with the most complete crystal structure available, or with a crystal structure of a homologous receptor. Purple spacefill residues on E2 showed <20% binding to CD81 when substituted to alanine (81, 105); residues with the same color and representation on CD81 showed reduced or eliminated binding to soluble E2 during random mutagenesis (100). Orange spacefill residues on LIMP-2 showed reduced binding to soluble E2 when mutated to a non-synonymous coding variant or the corresponding residue for mouse SR-BI (106, 107). Binding determinants of E2 to SR-BI are present in HVR1 (108), and are not present on the E2 crystal structure. Cyan spacefill residues on CD81 showed reduced association with CLDN1 when mutated to alanine (67), while cyan spacefill residues on CLDN1 showed either reduced binding to CD81 or decreased entry of HCVpp in alanine substitutions (67, 97, 109). PDB codes used are: 4MWF (E2), 5TCX (CD81), 4F7B (LIMP-2, representing SR-BI), and 4P79 (mouse claudin-15, representing claudin-1). SR-BI and CLDN1 have only moderate sequence identities to their structurally characterized homologs (LIMP-2 has 34% identity with SR-BI, mouse claudin-15 has 35% identity with human CLDN1), thus structures of these receptors may differ from the homologs shown.

Although many E1E2 modeling efforts have focused on antibody–antigen interactions or heterodimerization, some studies have examined the structures of host entry receptors and their interactions. The tetraspanin CD81 (92), scavenger receptor class B type I (SR-BI) (93), and tight junction proteins claudin-1 (CLDN1) (94) and occludin (OCLN) (95) represent the minimal set of HCV coreceptors and together are sufficient for HCV entry (96). Determinants of E1E2, glycoprotein–receptor, and receptor–receptor interactions are shown in Figure 3, summarizing current knowledge through high resolution or homologous protein structures that may inform prospective modeling studies. CD81 and SR-BI bind directly to E2 (92, 93) and CLDN1 associates with CD81 to permit HCV entry (97), but the basis of OCLN viral engagement is unknown. CD81 has been characterized the most among these receptors, due to its critical role in HCV entry, infection, and cell-to-cell transmission (98). Kong et al. modeled the CD81–E2 interface using restraints-guided docking using restraints-guided docking (47) with the HADDOCK modeling program (99), which incorporated mutagenesis data into structure prediction. The model was corroborated by a negative stain electron microscopy structure containing E2 and CD81 large extracellular loop (LEL) reported in the same study; the interface contained the CD81-LEL C and D helices, which are implicated in E2 binding (100). To validate this model experimentally, the authors generated E2 mutants based on their docking model that disrupted CD81 binding. A subsequent study (101) concentrated on the interface between CD81-LEL and antigenic domain D, using PEP-FOLD (91) to model the peptide and the AutoDock Vina program (102) for docking to a CD81-LEL crystal structure. CD81 MD have also explored CD81-LEL flexibility, and several crystal structures found pH-dependent conformational changes in these loops (103). The CD81-E2 interface could soon be resolved in greater detail through additional modeling or experimental studies, given that new CD81 crystal structures are available (103, 104) and that CD81 binding determinants on E1E2 have recently been fully delineated through global alanine scanning (81).

Although fewer modeling studies have focused on other HCV receptors, these provide important insights into the structure and recognition of these molecules. SR-BI does not have a reported X-ray structure, making its interactions with E2 relatively challenging to model with protein docking methods. However, the crystal structure of the closely related LIMP-2 (PDB code: 4F7B) led to a homology model of SR-BI, which was then used to elucidate the structural basis of its role in cholesterol uptake (66). Related scavenger receptor CD36 also has a crystal structure available (PDB code: 5LGD) (110), and was recently proposed as an additional coreceptor that binds E1 (111). Several studies have examined the structural determinants of the CLDN1–CD81 interface (97, 112, 113). *In silico* mutagenesis of this interface revealed key binding residues (67), and MD simulations of CLDN1 point mutations showed disruptions of receptor structure thought to diminish HCV entry (114). There is no reported X-ray crystal structure of CLDN1, but several claudin family members have solved structures (115, 116).

## Discussion

Given the numerous unknown aspects of the structural basis of HCV envelope glycoprotein assembly, as well as uncertainties regarding antibody and receptor recognition, there is a unique opportunity to leverage modern computational modeling and design algorithms to provide insights and testable mechanistic hypotheses for this system. Based on the challenges inherent in modeling this unique and dynamic viral envelope, future studies can utilize iterative experimental, and modeling approaches, where data-driven modeling is validated through experiments suggested by a model or sets of models. This paradigm has been utilized in previous studies to select and confirm models of antibody–antigen complexes (117, 118), as well as a modeled coiled coil assembly (119).

One additional area of recent interest has been the use of computational structure-based methods to design optimized protein and epitope-based immunogens for vaccines to better engage and elicit neutralizing antibodies, also known as “reverse vaccinology” (120). As seen for modeling, recent work has shown that iterative computational and experimental approaches are quite effective for vaccine design (121). Some have noted that HCV is a promising potential target for structure-based vaccine design (122), and early efforts have shown promise (15–17). Such work includes the design of scaffolded constructs based on the β hairpin form of antigenic domain E and an epitope from E1 (aa 314–324), and the display of these designs on protein nanoparticles, which showed maintained binding to the epitope-specific antibodies HCV1 and IGH526. In another study, a cyclic peptide design based on antigenic domain E, stabilized with a disulfide bond, was found to be immunogenic in mice; the X-ray structure of an induced murine antibody in complex with this design was determined (16), but no neutralizing antibodies were detected. A more recent vaccine design study reported two other cyclic antigenic domain E peptide designs, as well as a design of E2 with two copies of the antigenic domain E epitope based on structural similarity of a site on the E2 back layer to the β hairpin domain E structure (17). These designs elicited neutralizing antibodies in mice, but varied in H77 neutralization potency and showed limited response to the two non-H77 isolates tested (17). Follow-up studies as well as additional novel designs are needed to demonstrate the potential of rational vaccine design approaches for this virus. Furthermore, though cellular immunology is outside the scope of this review, fine mapping and molecular characterization of T cell epitopes may provide useful information to optimize vaccine constructs that will enhance or focus cellular immune responses, possibly in the context of a B-cell-based vaccine. The recently described structure of a T cell receptor engaging an immunodominant epitope from the HCV NS3 protein (123) is a compelling example for such a strategy.

The increasing application of powerful computational structural modeling techniques has led to a number of insights into HCV and its envelope glycoproteins. With the rapidly growing amount of data, including epitope mapping, structural characterization, and immune repertoire sequencing (124), there will be many opportunities to utilize these methods, to contribute further to the understanding of HCV immunogens, and to design an HCV vaccine. Centralized and up-to-date databases, resources, and standards for those focused on HCV research should facilitate these efforts. Effective resources may be analogous to a database developed for HIV bnAbs (125) or an existing database on HCV sequences and immunology (126). These resources will in turn permit the development of improved algorithms, more accurate models, and additional collaborative efforts focused on elucidating the native assembly and key features of the HCV envelope and eradicating HCV through an effective vaccine.

## Author Contributions

Both authors wrote and edited this work, and approved it for publication.

## Conflict of Interest Statement

The authors declare that the submitted work was carried out in the absence of any personal, professional, or financial relationships that could potentially be construed as a conflict of interest. The handling Editor declared a past co-authorship with one of the authors BP.
